# A novel pancreatic tumour and stellate cell 3D co-culture spheroid model

**DOI:** 10.1186/s12885-020-06867-5

**Published:** 2020-05-27

**Authors:** K. J. Norberg, X. Liu, C. Fernández Moro, C. Strell, S. Nania, M. Blümel, A. Balboni, B. Bozóky, R. L. Heuchel, J. M. Löhr

**Affiliations:** 1 Pancreas Cancer Research Lab, Department of Clinical Intervention and Technology (CLINTEC), Karolinska Institutet, Novum, floor 6, room 613, SE-141 86 Stockholm, Sweden; 2 Department of Laboratory Medicine (LabMed), Division of Pathology, Karolinska Institutet, Stockholm, Sweden; 3 Department of Clinical Pathology/Cytology, Karolinska University Hospital, Stockholm, Sweden; 4 Department of Cancer, Division of Upper GI, Karolinska University Hospital, Stockholm, Sweden

**Keywords:** PDAC, 3D cell culture, Virtual sorting, Gene expression, Real time RT-PCR

## Abstract

**Background:**

Pancreatic ductal adenocarcinoma is a devastating disease with poor outcome, generally characterized by an excessive stroma component. The purpose of this study was to develop a simple and reproducible in vitro 3D-assay employing the main constituents of pancreatic ductal adenocarcinoma, namely pancreatic stellate and cancer cells.

**Method:**

A spheroid assay, directly co-culturing human pancreatic stellate cells with human pancreatic tumour cells in 3D was established and characterized by electron microscopy, immunohistochemistry and real-time RT-PCR. In order to facilitate the cell type-specific crosstalk analysis by real-time RT-PCR, we developed a novel in vitro 3D co-culture model, where the participating cell types were from different species, human and mouse, respectively. Using species-specific PCR primers, we were able to investigate the crosstalk between stromal and cancer cells without previous cell separation and sorting.

**Results:**

We found clear evidence for mutual influence, such as increased proliferation and a shift towards a more mesenchymal phenotype in cancer cells and an activation of pancreatic stellate cells towards the myofibroblast phenotype. Using a heterospecies approach, which we coined virtual sorting, confirmed the findings we made initially in the human-human spheroids.

**Conclusions:**

We developed and characterized different easy to set up 3D models to investigate the crosstalk between cancer and stroma cells for pancreatic cancer.

## Background

Poor response to therapy and a dismal prognosis are the hallmarks of pancreatic ductal adenocarcinoma (PDAC), which constitutes 2.5% of the worldwide cancer incidence, yet 4.5% of the mortality [1]. In spite of increasing research efforts in recent years, the mortality rate of PDAC has remained high while other cancer mortality has significantly decreased. Pancreatic cancer remains the fourth leading cause of cancer-related death in the world, and unless a remarkable break-through is soon achieved, it will continue to climb to number two on the list of cancer-related cause of death [2].

PDAC is generally characterized by a particularly dense and fibrotic stroma, largely composed by pancreatic stellate cells (PSCs). When activated, the PSCs express α-smooth muscle actin (ASMA/ACTA2) and induce extensive desmoplasia by secreting profuse amounts of extracellular matrix (ECM) proteins [3] such as fibronectin and collagen. Transforming growth factor β (TGF-β) is known to moderate fibroblast phenotype and function, inducing myofibroblast transdifferentiation, while increasing stromal stimulation and ECM production [4]. Importantly, the PSCs also communicate extensively with both tumour cells as well as other stromal cells, collectively supporting tumour progression through modification of various pathways [3]. The function of the PDAC stroma in PDAC chemo-resistance has been widely debated [5]. It has been demonstrated to act as a host defence against cancer and block chemotherapeutic drugs from reaching the tumour [6]. Depletion of the stroma by the sonic hedgehog inhibitor saridegib (IPI-926, Infinity Pharmaceuticals) in clinical trials has, however, proven to produce more aggressive tumours and lower survival, which was later supported by two independent animal studies [7, 8]. More recently, a re-education instead of depletion of the tumour stroma has been suggested. For instance, Sherman et al. specifically de-activated PSCs by activating vitamin D receptor signalling resulting in stromal reprogramming and increased chemosensitivity [9]. Nevertheless, a deeper understanding of the PDAC stroma and its communication with the tumour cells, is desperately needed [10].

Monolayer growth of cells in traditional cell culture differs from the three-dimensional (3D) growth of solid tumours in vivo in several important ways. The cell growth in 3D causes gradients of oxygen, nutrients as well as waste products etc. It also has been generally accepted that 3D cell-cell interactions influence cell signalling in response to soluble factors, something that greatly affects cell function [11]. Various 3D models of human cancer have been developed [12, 13], including multi-layered tumour cell cultures, tumour slices [14], organoids, 3D cultures with reconstituted basement membranes and spherical cancer models [15]. To date, there are various 3D-culture models of pancreatic cancer, counting numerous multicellular tumour spheroid (MCTS) models [16–19], including our previously established 3D spheroid mono-culture model, characterized by a higher ECM expression and significantly increased chemo-resistance compared to cells cultured in monolayers [20]. Although organoids and organotypic multicellular spheroids derive from and are closer to real tumours, simpler models such as MCTS have the advantage of ease of maintenance and the possibility for high throughput drug screening as well as genetic manipulation of the cells [11]. Extending MCTS and other matrix 3D models to allow for studies of tumour-stromal cross-talk, there are now e.g. tumour spheroid models of cancer cells and fibroblasts in lung cancer and cervical carcinoma [21], melanoma [22], breast [23, 24], colorectal [25, 26], liver [27] as well as pancreatic cancer [28]. Although more complex models with multiple cell lines are desirable to answer certain types of questions, there is still a need for simpler co-culture models with only two cell types, where direct cell cross-talk can be more easily investigated. In addition, we believe that there is great diversity in behaviour amongst both tumour cells and the various kinds of fibroblasts and PSCs [29]. To this end, numerous models using different types of cells will be needed to advance the field, where the model of choice will be determined by the question being studied. Here, we extensively characterized a novel scaffold-free 3D spheroid model of direct PDAC and PSC co-cultures, with two different human tumour cell lines (Panc1 and HPAFII cells) co-cultured with human PSCs focusing on the study of cellular cross-talk. We also developed a novel approach of investigating cell-type specific gene expression from non-sorted, intact spheroids, named virtual sorting, by utilizing cross-species co-cultures in combination with species-specific primers.

## Methods

### Cell culture

Panc1 (CRL-1997) and HPAFII (Cat. no. 87092802) cells were purchased from ATCC and ECACC, respectively, and are well characterized [30]. The human pancreatic stellate cells (hPSC) were isolated in house [31]. The KPCT 86–2 cell line was isolated in-house from a *Kras*^*LSL-G12D/+*^*;Trp53*^*LSL-R172H/+*^*;Pdx-Cre* (KPC) mouse [32] mated to the tdTomato allele (B6.Cg-*Gt (ROSA)26Sor*^*tm9(CAG-tdTomato)Hze*^/J) [33]. The immortalized mouse pancreatic stellate cell line clone 3 (imPSCc3; in text and figures referred to as mPSC) was a kind gift from Dr. Raul Urrutia and Dr. Angela Mathison at the Mayo Clinic College of Medicine, Rochester, Minn, USA [34]. All cell lines were cultured under standard culture conditions (5% CO_2,_ at 37 °C) in culture media (Panc1, hPSC, KPCT 86–2 and imPSCc3 in DMEM/F12 media and HPAFII in RPMI-1640) supplemented with 10% FBS (according to ATCC recommendations) and 0,5% penicillin/streptomycin. All cells were tested negative for mycoplasma (MycoAlertTM PLUS Mycoplasma Detection Kit, LT07–705, Lonza, Switzerland) and for active retrovirus (Reverse transcriptase assay, colorimetric, 11468120910, Roche, purchased through Sigma Aldrich, Sweden).

### Spheroid preparation

Both human and mouse PDAC tumour and pancreatic stellate cells were seeded alone or in co-culture (ratio: 1:1), total concentration 2500 cells/well in non-cell culture treated round bottom 96-well plates (Falcon, BD NJ, USA). Cells were seeded in culture media with a final concentration of 0.24% methylcellulose to support self-aggregation [20, 35]. When setting up spheroid experiments, all cells including HPAFII cells were seeded in DMEM/F12 media. After the indicated number of days, the formed spheroid cultures were collected and processed for downstream purposes.

### Transmission electron microscopy (TEM)

Mono- and co-culture spheroids were collected with a 1 ml pipette tip into a 15 ml tube. The spheroids were washed once in PBS before being fixed in 2,5% glutaraldehyde in 0,1 M PBS. Imaging was performed on a Tecnai 12 Spirit Bio TWIN transmission electron microscope (Fei Company, Eindhoven, The Netherlands) at the Central Electron Microscopy Unit of Karolinska Institutet. Three individual spheroids were analysed for each type of spheroid in each experiment. For each spheroid, one overview low magnification image was taken, covering both peripheral and central areas. An additional 20 images per spheroid were then taken, 10 images peripherally and another 10 images centrally. These 10 images covered 5 different areas, with one lower magnification and one higher magnification image at each site.

### Spheroid embedding and histology

Spheroid mono- and co-cultures were fixed in buffered 4% paraformaldehyde for 24 h at room temperature before changing into 70% ethanol at 4 °C. The spheroids were embedded in HistoGel (ThermoFisher Scientific, HG-4000-012,), according to a modified protocol [36]. A tube of HistoGel was heated up in a water filled beaker in the microwave until the gel was liquefied. The spheroids were transferred into a corner of a tilted biopsy cryomould (Tissue-Tek Cryomould #4565; 10x10x5mm), 100 μl of liquefied HistoGel was then added to the mould. The mould was kept tilted until the HistoGel had solidified. Then the moulds were filled up with another 350 μl of liquid HistoGel. The solidified gel block containing the spheroids, was gently pushed into a larger biopsy cassette, and transferred to 70% ethanol for paraffin-embedding. Then, spheroids were sectioned (4 μm thick) before staining with haematoxylin-eosin or immunohistochemistry performed by the routine histopathology laboratory at Karolinska University Hospital.

### Immunohistochemical staining and analysis

Immunohistochemical staining was performed using a Leica BOND III (Leica Biosystems Melbourne Pty Ltd., Mount Waverly, Australia) or a Ventana Benchmark Ultra (Ventana Medical Systems Inc., Tucson, Arizona, United States) automated immunostainer, with the panel of antibody markers given in supplementary Table 1. MKI67 and HMGA2 were combined with CK19 by multiplex immunohistochemistry, in order to discriminate their expression among tumour cells (CK19^+^) and PSCs (CK19^−^), respectively [37].

Histological slides were digitalized with a 3D Histech Panoramic SCAN II slide scanner. Quantitation of immunohistochemical markers was performed on the whole slide images using QuPath v1. 3[38]. Initially, spheroids were detected by applying tissue detection. Samples that were too large, representing single areas containing multiple spheroids, or too small, representing peripherally cut spheroids, were excluded from that analysis. Further inaccurately detected regions were excluded, or manually corrected. MKI67 was quantified in the tumour cells (CK19^+^) and the PSCs (CK19^−^) by means of the threshold of nuclear DAB, following the method of watershed cell detection (https://www.pyimagesearch.com/2015/11/02/watershed-opencv/). E-cadherin and WT1 were quantified globally for all cells in the spheroids by thresholds of cytoplasmic and whole cell DAB, respectively. The total number and the percentage of positive tumour cells and PSCs (MKI67), or the global percentage of positive cells (E-cadherin, WT1), were obtained for each spheroid and then averaged for each slide. Standard deviations were calculated and are shown as error bars. CD10 and vimentin were assessed visually and not digitally quantitated due to their diffusely positive or negative expression.

### Virtual sorting

Species-specific primers were designed in areas genetically diverse in the mouse and human homologue genes, using NCBI Blast (https://blast.ncbi.nlm.nih.gov/). These were tested by real time RT-PCR on a test panel including human tumour cells (Panc1), human PSCs (hPSC), mouse tumour cells (KPCT 86–2) and mouse PSCs (imPSCc3). The products of the PCR reactions were run on a 2% agarose gel (TAE buffer), in order to ensure species specificity (Suppl. Fig. 6). Species-specific primer pairs were then used for hetero-species heterospheroid gene expression analysis.

### Real time RT-PCR assays

Spheroids from one 96-well plate per spheroid type were collected, washed twice with cold PBS, spun down and processed for total RNA isolation using the RNeasy Kit (Qiagen, 74,104). 1 μg of total RNA was retro-transcribed using the iScript cDNA Synthesis kit (Bio-Rad, 1,708,891). SYBR-Green technology (ThermoFisher Scientific, K0243) was used for real time RT-PCR using the following amplification program: initial denaturation 10 min at 95 °C, 40 cycles of 15 s at 95 °C and 1 min at 60 °C. All SYBR-Green assays were run in triplicates with human or mouse *Rpl13a* as housekeeping gene. Delta CT values were used for statistical analyses with the Student’s *t*-test (2-sided, individual samples) for a minimum of 3 independent experiments. Average mRNA expression, normalized to day 3 tumour cell mono-spheroids, from the replicate experiments are shown in the figures, with 95% confidence intervals as the standard deviation bars.

## Results

### Electron microscopy revealed healthy spheroid cultures

**Fig. 1:**
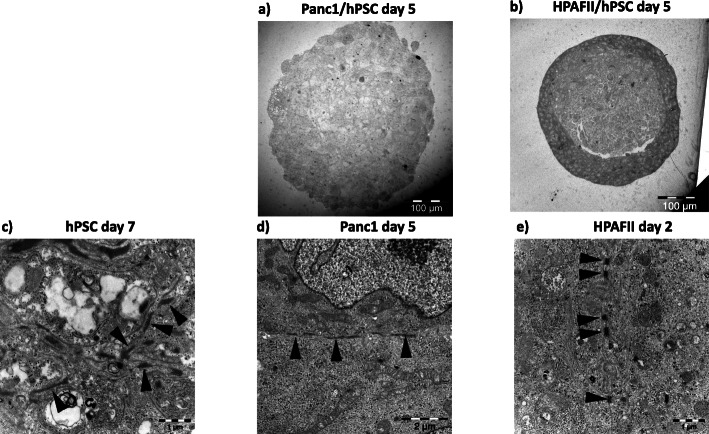
Transmission electron microscopy of spheroid sections. Central section giving an overview of representative Panc1/hPSC (**a**) and HPAFII/hPSC (**b**) spheroids after 5 days of culture. A day 7 PSC mono-spheroid containing immature desmosomes (arrow heads, **c**). Panc1 cells in a mono-spheroid from day 5 with immature desmosome-like connections (arrow heads, **d**). A day 2 HPAFII mono-spheroid showing cytoplasm and developed desmosomes (arrow heads, e)

In order to get a general impression on the status and morphology of the spheroids, we performed transmission electron microscopy revealing generally healthy spheroid cultures, with very few apoptotic and necrotic cells, as well as moderate extracellular matrix, in both Panc1/PSC and HPAFII/PSC mono- and heterospheroids (Fig. 1a, b and Suppl. Fig. 1a). Striking is the clear separation between the HPAFII cells and PSCs, whereas such a distinction cannot be detected in the Panc1/PSC heterospheroids (Fig. 1a, b).

In PSC mono-cultures, outer layer cells were flatter and had more contact surface between themselves and cells of the next layer, compared to cells more central in the spheroids (Suppl. Fig. 1b). The surface layer of cells displayed more microfilaments reaching outwards. Desmosome-like contact surfaces were detected, seen as black bands in Fig. 1c. Similar contact surfaces were also observed in the central part of HPAFII/PSC heterospheroids. Almost no extracellular matrix was seen in the PSC mono-cultures.

Panc1 mono-cultures displayed only scarce extracellular matrix. Cell-cell contacts (Fig. 1d) looked similar to those in PSC mono-cultures, although the packaging of the cells was less compact (Suppl. Fig. 1a). The Panc1/PSC heterospheroids presented with some extracellular matrix, including some collagen fibres (Suppl. Fig. 1c). Overall, the co-cultures had rather small amounts of extracellular matrix. The desmosome-like cell-cell contacts did not mature further upon coculture.

HPAFII mono-spheroids presented as doughnut structure in central sections (Suppl. Fig. 1a). The differentiated epithelial cells contained plenty organelles with good ultrastructure and large amounts of well-developed desmosomes, at all timepoints bestowing them with a compact appearance (Fig. 1e and data not shown). Peripheral cells were rounded and slightly polarized, with the cytoplasm outwards and the nucleus oriented towards the center. These spheroids displayed fair amounts of superficial microvilli, with glycocalyx (“sugars”) similar to those found on intestinal cells. Some larger vacuoles were detected, containing some extracellular matrix and dressed with glycocalyx-coated microvilli on the luminal side (Suppl. Fig. 1d).

In the HPAFII/PSC co-cultures, cells were round and there were some microvilli with glycocalyx. The microvilli decreased with time also in these cultures, mainly from day 5 to 7. The peripheral cells showed more detailed ultrastructure than the centrally located lighter grey appearing cells, which had a looser appearance (Fig. 1b). Some few vacuoles with microvilli were detected. The cell-cell contacts in the co-cultures were generally well evolved. Both non-developed and developed desmosomes were seen, the latter ones mainly in the outer shell structure.

### Panc1 cells co-cultured with hPSCs proliferate more and lack E-cadherin

**Fig. 2:**
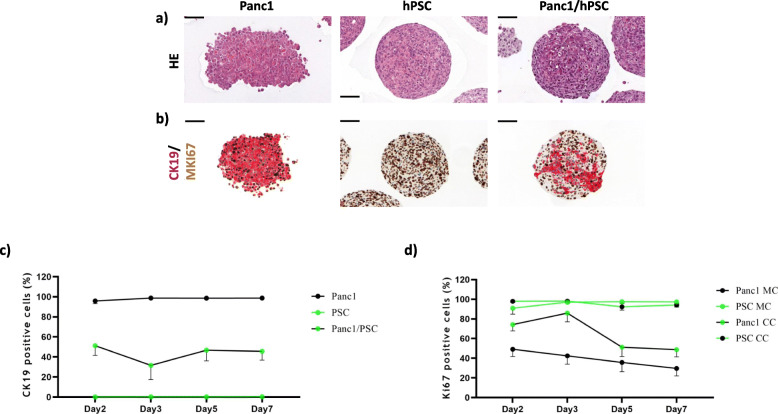
Histological and immunohistochemical characterization of Panc1 and hPSC mono- and heterospheroids cultured for 5 days. Haematoxylin and eosin-stained central sections of mono- and co-cultured Panc1/hPSC spheroids (**a**). Immunohistochemical double-staining for the epithelial marker CK19 (red) and the proliferation marker MKI67 (brown) (**b**). CK19/MKI67 double staining was also used to determine the percentage of Panc1 cells (CK19^+^)/hPSC cells (CK19^−^) (**c**) and the percentage of proliferating (MKI67^+^) Panc1 and hPSC cells (**d**) in mono- and heterospheroids, corresponding to mono-culture (MC) and co-culture (CC), respectively, over a period of 7 days. Black scale bars in **a**) and **b**) correspond to 100 μm

**Fig. 3:**
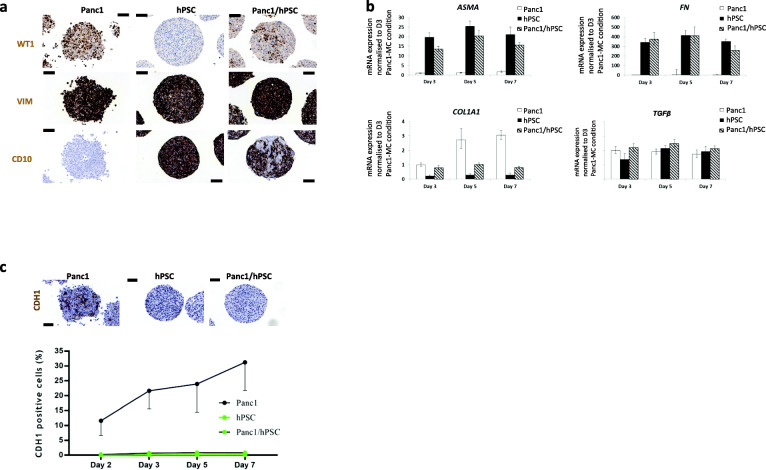
Immunohistochemical characterisation and expression analyses of Panc1/hPSC mono- and heterospheroids. Immunohistochemical staining for WT1 (Wilms tumour 1), VIM (vimentin) and CD10 (MME, membrane metalloendopeptidase) of Panc1 and hPSC mono- and heterospheroids cultured for 5 days (**a**)**.** Normalized mRNA expressions of *ASMA* (ACTA2, actin alpha 2, smooth muscle), *COL1A1* (collagen 1 type A1), *FN* (fibronectin) and *TGFβ1* (transforming growth factor beta 1) are shown from spheroid cultures at the indicated time points. All values have been normalized to the expression of the individual genes in Panc1 mono-cultures at day 3 (**b**). Representative example of an E-cadherin (CDH1) protein staining (brown colour) from day 5 spheroids and the detailed quantification of CDH1-positive cells over a time period of 7 days (**c**). Black scale bars in **a**) and **c**) correspond to 100 μm

Morphological analysis of hematoxylin/eosin stains of our human-human tumour cell and pancreatic stellate cell spheroid cultures confirmed the TEM observations demonstrating healthy cells with negligible apoptosis and necrosis accounting to about 1 to 2% of all cells in the different spheroid types. There was a lack of visible division between the tumour and stroma cells within the co-cultured spheroids (Fig. 2a). CK19/MKI67 double staining indicated the distribution of epithelial tumour cells (CK19^+^) and hPSCs (CK19^−^), as well as their proliferative capacity, indicated by MKI67-positiveness (Fig. 2b). Quantification of CK19^+^ cells in the mono-cultured spheroids (Fig. 2c) confirmed the uniform presence of CK19 positivity in all epithelial Panc1 cells as well as its complete absence in the hPSCs. CK19 staining of the co-culture spheroids indicated also some fluctuation in the distribution of the two cell types over time, stabilizing at later time points at around 50:50. Quantification of MKI67^+^ cells in mono- as well as co-cultures (Fig. 2d) showed that almost all hPSCs were proliferating. Interestingly, the results also revealed that co-culture with hPSCs increased the percentage of MKI67^+^ proliferative Panc1 cells (Fig. 2d). Staining of another epithelial marker (WT1) validated the epithelial specificity of the CK19 staining (Fig. 3a). Both Panc1 cells and hPSCs stained positive for vimentin, whereas hPSCs but not Panc1 cells were found positive for CD10 (Fig. 3a).

Real time PCR of spheroid preparations confirmed the mRNA expression of epithelial markers CK19 and WT1 in Panc1 cells but not hPSCs, and co-cultures indicated a reduction of mRNA reflecting the expected ratio of both cell types (Suppl. Fig. 2a-b). Likewise, CD10 mRNA was exclusively detected in hPSCs but not in Panc1 cells (Suppl. Fig. 2c). CK19 expression in Panc1 mono-cultures decreased, whereas CD10 expression in hPSC mono-cultures increased over time. The mRNA of ASMA (ACTA2) and the extracellular matrix protein fibronectin (FN), both markers of activated stellate cells were expressed in hPSCs but not in Panc1 mono-cultures and their expression levels relatively increased upon co-culture, since the expression stems only from around 40–50% of the cells in the co-culture (Fig. 3b). Collagen type I mRNA expression increased in Panc1 mono-cultures over time, whereas expression in hPSC mono-cultures and co-cultures remained stable (Fig. 3b). TGFβ1 mRNA measurement suggested a trend towards slightly increased expression in co-cultured compared to mono-cultured spheroids (Fig. 3b).

As expected, the epithelial marker E-cadherin (CDH1) was partially expressed in Panc1 but not hPSC mono-cultures. Interestingly, CDH1 protein seemed almost completely suppressed in the co-cultures (Fig. 3c). Quantification of the protein staining also revealed that CDH1 expression increased in Panc1 mono-cultures over time (Fig. 3c).

### HPAFII co-cultures distinctly differ from Panc1 co-cultures in proliferation and expression

**Fig. 4:**
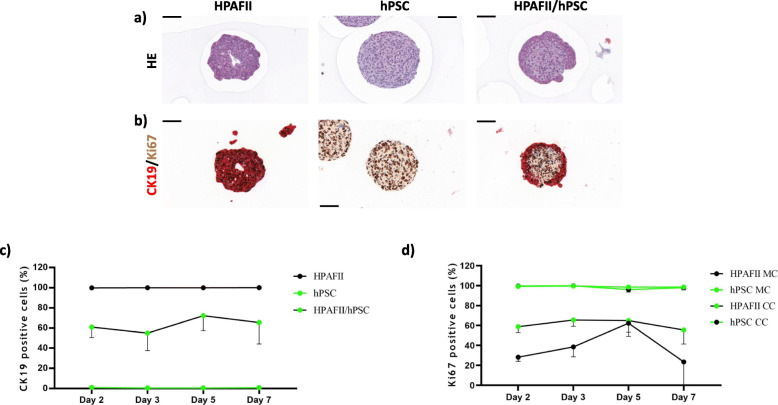
Histological and immunohistochemical characterization of HPAFII and hPSC mono- and heterospheroids cultured for 5 days. Haematoxylin and eosin-stained central sections of mono- and co-cultured HPAFII/hPSC spheroids (**a**). Immunohistochemical double-staining for the epithelial marker CK19 (red) and the proliferation marker MKI67 (brown) (**b**). CK19/MKI67 double staining was also used to determine the percentage of HPAFII cells (CK19^+^)/hPCS cells (CK19^−^) (**c**) and the percentage of proliferating (MKI67^+^) HPAFII and hPSC cells (**d**) in mono- and heterospheroids, corresponding to mono-culture (MC) and co-culture (CC), respectively, over a period of 7 days. Black scale bars in **a**) and **b**) correspond to 200 μm

Hematoxilin/eosin staining confirmed that HPAFII spheroids form hollow, glandular-like structures, whereas PSC spheroids were solid. In HPAFII/hPSC heterospheroids, we found one cell type forming a ring around a core of slightly brighter stained cells (Fig. 4a) confirming the compartmentalisation already observed in the TEM analysis (Fig. 1b). CK19/MKI67 double staining identified the outer ring structure in heterospheroids as HPAFII cells (CK19^+^) and the core as hPSCs (CK19^−^) (Fig. 4b). MKI67 labelled all proliferating cells (MKI67^+^) independent of spheroid type (Fig. 4b). CK19 quantification (Fig. 4c) illustrated a larger number of HPAFII cells than hPSCs in the co-cultures with a further slight increase of HPAFII cells at later time points. Quantification of MKI67^+^ cells (Fig. 4d) showed that virtually all hPSCs in both mono- and HPAFII co-cultures were proliferating. Importantly it also demonstrated that the proportion of proliferating HPAFII cells was generally increased by the co-culture with hPSCs.

The epithelial marker WT1 was not expressed in HPAFII cells (data not shown), whereas hPSCs, but not HPAFII cells presented positive for vimentin and CD10 (Suppl. Fig. 4a b), once more confirming that the core compartment of these heterospheroids is made up of PSCs. The epithelial marker CK19 was also expressed on the mRNA level in HPAFII cells, but not hPSCs (Suppl. Fig. 3a). Furthermore, the CK19 mRNA data from the co-cultures supported the slight increase in HPAFII cell ratio over time observed in the immunohistochemistry for CK19 from Fig. 4c. WT1 was not expressed in HPAFII cells, neither at mRNA nor at protein level (data not shown). CD10 mRNA was strongly expressed in the hPSCs, but not in the HPAFII cells (Suppl. Fig. 3b), in line with protein data (Suppl. Fig. 4b). ASMA, fibronectin and collagen type I were all expressed in hPSCs, but not in HPAFII mono-cultures. Their expression seemed increased upon co-culture (Suppl. Fig. 4c), always assuming that the co-cultures contained 40% or less PSCs (Suppl. Fig. 4c). TGFβ1 mRNA was found to be expressed in both HPAFII and hPSC mono-cultures and clearly increased in the day 7 co-cultures compared to the individual mono-cultures (Suppl. Fig. 4c).

E-cadherin (CDH1) was found highly expressed in HPAFII but not hPSC cells on the protein- (Suppl. Fig. 4d) as well as on mRNA level (data not shown). Quantification of the immunohistochemistry indicated a slight increase in the proportion of CDH1 expressing HPAFII cells at later time points (Suppl. Fig. 4d).

### Virtual sorting of spheroids made up of cells from different species facilitated stromal epithelial crosstalk analysis

**Fig. 5:**
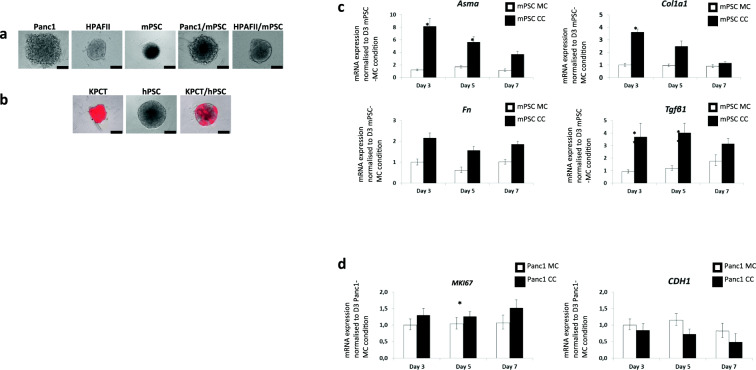
Spheroid cultures used for virtual sorting. Bright field (**a**) and combined bright field fluorescence photographs (**b**) of mono- and heterospheroids from day 5 of the indicated cell lines are shown. Panc1 and HPAFII are human tumour cell lines, mPSC is an immortalized mouse pancreatic stellate cell line. KPCT is a pancreatic cancer cell line established from a *Kras*^*LSL-G12D/+*^*;Trp53*^*LSL-R172H/+*^*;Pdx-Cre* (KPC) mouse mated to a mouse with the tdTomato allele (B6.Cg-*Gt (ROSA)26Sor*^*tm9(CAG-tdTomato)Hze*^/J) and hPSC is a human pancreatic stellate cell line. Virtual sorting real time PCR shows cell type- and species-specific mRNA expression in Panc1 and mPSC mono- and heterospheroids. The mRNA expression of *Asma (Acta2)*, *Col1a1*, *Fn* and *TGFβ*_*1*_ (**c**) and *MKI67* and *CDH1* (**d**) are depicted over a time period of 7 days. The values were normalized to day 3 mPSC- (**c**) and Panc1 mono-spheroids/−cultures (MC) (**d**), respectively. *p*-values were as follows: *, *p* < 0.05; **, *p* < 0.01. Black scale bars in a) and b) correspond to 200 μm

We previously determined cell-type specific mRNA expression by real time PCR after sorting the cells with fluorescence activated cell sorting (FACS ) [35]. During this procedure, we experienced over-proportional cell loss. Therefore, we developed a novel method to determine cell-type specific gene expression directly from non-dissociated, intact spheroids. This was achieved by culturing spheroids of human tumour and mouse stellate cells in combination with individually designed species-specific primers for real-time PCR. Human and mouse mono- and co-cultures of all cell lines used are shown in Fig. 5a, b. Real time PCR products were analysed on a 2% agarose gel, to test amplicon length and species-specificity of the primers (Suppl. Fig. 6).

Virtual sorting performed on spheroids of Panc1 cells, mouse PSCs (mPSC) and mixtures thereof convincingly demonstrated the activation of the stellate cells by the co-cultured Panc1 cells through significant upregulation of *Acta2* (Asma), *Col1a1* (collagen-1a1), *Fn* (fibronectin) and *Tgfb1* (transforming growth factor-β1) in the PSCs (Fig. 5c). Furthermore, a trend of increased proliferation (*MKI67*) of Panc1 cells upon co-culture could be observed (Fig. 5d). *Cdh1* mRNA was slightly lower expressed in co-cultured compared to mono-cultured Panc1 cells (Fig. 5d).

**Fig. 6:**
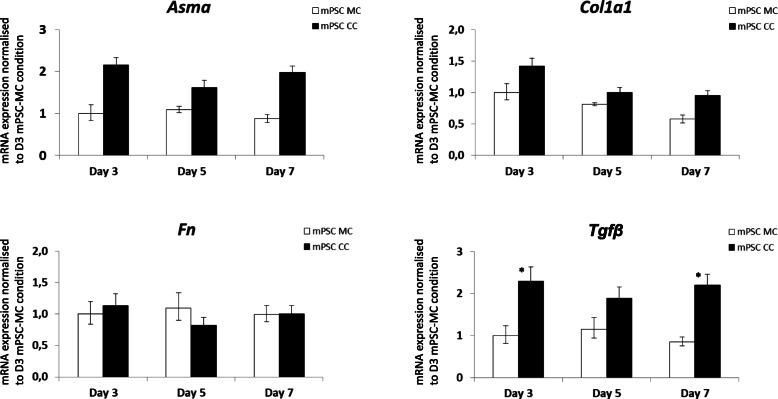
Virtual sorting rtPCR identified cell type and species-specific mRNA expression in mPSC mono- and co-cultures with HPAFII. The mRNA expression of mPSCs in mono- and heterospheroids of *Asma*, *Col1a1*, *Fn* and *Tgfβ*_*1*_ is depicted over a time period of 7 days. All expression values were normalized to mPSC mono-spheroids/−cultures (MC) at day 3. p-values were as follows: *, *p* < 0.05

The virtual sorting method on HPAFII and mPSC cells likewise indicated an activation of the HPAFII co-cultured mPSCs (Fig. 6), albeit slightly weaker, while *Fn* mRNA expression was not affected.

**Fig. 7:**
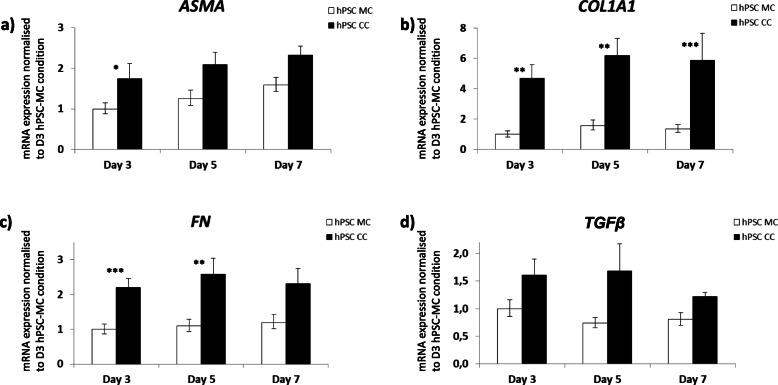
Virtual sorting rtPCR identified cell type- and species-specific mRNA expression in hPSC mono- and co-cultures with the murine pancreatic cancer cell line KPCT. mRNA expression of *ASMA*, *COL1A1*, *FN* and *TGFβ1* from hPSCs derived from mono- and heterospheroids are depicted over a time period of 7 days . All expression values were normalized to hPSC mono-spheroids/−cultures (MC) at day 3. *p*-values were as follows: *, *p* < 0.05; **, *p* < 0.01

In order to test the versatility of the mixed species approach further, we also cultured mouse KPCT tumour cells and human PSCs (Fig. 5b). Also, the human PSCs were activated by the co-cultured mouse tumour cells as indicated by increased *ACTA2*, *COL1A1*, *FN* and *TGFB1* mRNA levels (Fig. 7).

## Discussion

Since PDAC imposes a highly unmet medical need, studying the intimate relation between its two principal cellular components, tumour cells and PSCs, is pivotal for the understanding of the disease as well as a basis for possible therapy. We provide here a simple, reproducible (Suppl. Fig. 5) and scaffold-free, i.e. no Matrigel, collagen or other biomatrices, 3D model allowing for the separate analysis of the individual cellular compartments from the intact spheroid. We thoroughly characterized and validated 3D co-culture models of Panc1 or HPAFII PDAC cells with an immortalized human PSC line (hPSC) [31]. Interestingly, but not totally unexpected, the two models distinctly differ, most probably due to the different phenotypes of the participating tumour cells [30]. Panc1 cells are classified as quasi-mesenchymal compared to the more classical, epithelial HPAFII cells [39]. Panc1 originates from a grade 3 tumour and is poorly differentiated and more “EMT-ish”, whereas the HPAFII cell line was derived from a grade 2, moderately differentiated tumour [30]. Panc1 but not HPAFII cells stained positive for vimentin (Fig. 3a and Suppl. Fig. 4a). Quantification of E-cadherin staining in the Panc1/hPSC (Fig. 3c) and HPAFII/hPSC (Suppl. Fig. 4d) mono- and heterospheroids demonstrated a strong epithelial phenotype of HPAFII but not Panc1 cells. The staining also revealed an increase of the E-cadherin expression in cell clusters inside the Panc1 mono-spheroids over time, most probably reflecting an ongoing compaction process. On the other hand, the presence of the PSCs seemed to completely suppress E-cadherin expression in the Panc1 cells, possibly pushing them even further into a mesenchymal phenotype. An increase in proliferation and shift towards a more mesenchymal phenotype has also been detected in a 3D lung cancer co-culture model [40]. The epithelial HPAFII cells strongly expressed E-cadherin in both, spheroid mono- and co-cultures. In stark contrast to the intermixed Panc1 and PSC cells in the co-culture condition, the HPAFII and PSC cells are clearly separated. We can only speculate that this segregation could be due to stronger homophilic than heterophilic interactions for HPAFII and possibly also PSCs. For strong homophilic interactions of the HPAFII cells speaks the high expression of E-cadherin (Suppl. Fig. 4d).

The electron microscopy analysis (Fig. 1 and Suppl. Fig. 1) also supports a stronger epithelial phenotype of the HPAFII compared to the Panc1 cells. HPAFII cells were connected through fully developed desmosomes in both mono- and heterospheroids, whereas only very few immature desmosome-like connections were seen between Panc1 cells. Desmosomes confer strong connections and predominantly occur between epithelial cells, ensuring physical strength. In general, TEM confirmed the healthiness of the 3D spheroid model without considerable signs of apoptotic or necrotic cells (Fig. 1 and Suppl. Fig. 1). Here, it should be noted that on day 5, our spheroids, with the exception of the more loosely packed Panc1 monospheroids, were below a diameter of 500 μm, the size which usually results in central necrotic cells due to limited diffusion of nutrients and oxygen (Suppl. Fig. 5).

As seen in Figs. 2 and 4b-c, CK19 antibodies undoubtedly mark only the epithelial tumour cells (Panc1, HPAFII) but not the hPSCs, thus making it possible to separate the two cell types in the co-cultures and to determine their proliferation by MKI67 double-staining (Figs. 2 and 4b, d). MKI67 protein quantitation by IHC revealed a generally increased proliferation in heterospheroid tumour cells, demonstrating induction by the stromal compartment (PSCs).

We developed the virtual sorting method for determination of cell type specific gene expression, for a number of reasons. Although FACS is a very useful technique, it requires the spheroids to first be dissociated into single cells. This turned out to be very inefficient for our spheroid cultures and naturally a significant number of cells were also lost during the actual sorting process, especially in the co-culture spheroids. This very lengthy sample prepping and preparative sorting time could introduce cellular stress, affecting gene expression in unwanted ways [41]. The great advantage of the virtual sorting method appears when genes that are expressed in both participating cell types are to be investigated, like MKI67/Mki67, COL1A1/Col1a1 or TGFβ/Tgfβ. The virtual sorting approach has two drawbacks. Some signalling molecules, e.g. the species-specific interferons are not recognized by the second participating cell type and sometimes it is difficult to select species-specific gene primers. In addition, it is desirable that the PSCs from different species behave in a comparable manner. As we have learned, not only tumour cells lines but also pancreatic stellate cells and CAFs differ significantly in their characteristics, also in the same tumour [29, 42]. However, in the framework of our experiments, we could not detect major differences between mouse and human PSCs.

In this study, using the novel method of virtual sorting, we successfully confirmed several observations, we initially made in our human-human heterospheroid cell cultures. Virtual sorting of Panc1 and mPSC co-culture spheroids, for example, confirmed the already mentioned increase in MKI67 expression in Panc1 cells, as well as a lower expression of CDH1 in the co-cultured compared to mono-cultured Panc1 cells (Fig. 5d). The method also confirmed the activation of mouse PSCs upon co-culture with tumour cells, through mRNA expression of the classical activation markers *Asma/Acta*, *fibronectin (Fn)*, *collagen-1a1 (Col1a1)* and *Tgfβ1* (Figs. 5c, and 6]. Collagen type I mRNA expression increased in the Panc1 mono-cultures over time (Fig. 3b) and seemed much higher expressed in Panc1 cells than in mono-cultured PSCs. Interpretation for expression changes between pure human mono- and co-cultures were difficult, however the virtual sorting of Panc1 cells and mPSCs suggested a slight increase of collagen1a1 mRNA in co-cultured mPSCs (Fig. 5c). Taken together, these increases in activation markers for PSCs and extracellular matrix genes were a clear evidence of cross-talk between the co-cultivated cell types and demonstrated classical activation of the PSCs. The fact that the PSCs had a very high proliferative index (Figs.2, and 4d), independent of mono- or heteroculture condition, might be due to that these cells have been immortalized [31], or that the cells in the spheroids are not “locked” in a rigid collagen matrix like in tumour tissue. Reports about the proliferation rate of PSCs in PDAC are scarce and vary [43–45], thus need further investigation. The problem of increased proliferation of immortalized fibroblasts in vitro has been observed also in a 3D lung cancer co-culture model [40] and has to be considered when planning drug screening assays for cytotoxic compounds. On the other hand, primary fibroblasts seem to get lost after only a few days of 3D co-culture with Panc1 cells, limiting the usefulness of this approach [46].

CD10^+^ stroma has been found in breast [47], gastric [48] and colon cancer, and was shown to be a prognostic marker associated with more aggressive disease. CD10^+^ PSCs in pancreatic cancer were shown to enhance disease progression [42]. Those CD10^+^ PSCs were however strongly ASMA^+^, expressing the protein as shown by both immunohistochemistry and immunofluorescent staining. Our immortalized human PSC line is negative for ASMA on the protein level, and these cells have recently been shown to not enhance migration or gemcitabine resistance [29]. This may indicate that dual positivity would be needed, and again shows the need for a greater understanding of the different populations of stellate cells and the complex cross-talk between these populations and various tumour cells.

In our study however, the 3D aspect adds another dimension. As shown, 3D co-culturing diminished E-cadherin expression in Panc1 cells. In a previous study, we demonstrated an increase of HMGA2, a marker of lower overall survival and increased migration and metastasis in the co-cultured vs mono-cultured Panc1 cells [35]. Both the loss of *CDH1* and the increase of HMGA2 are characteristic for cells undergoing epithelial-to mesenchymal transition (EMT) [49].

We strongly believe that a wider variety of both stromal cell lines and primary stromal cells, as well as co-culture models between tumour cells and additional cell types from their microenvironment are needed in order to investigate numerous relevant questions. Our heterospecies heterospheroid approach allows the combination of any two “compatible” cell types available from different species. Addition of further cell type/species is theoretically possible but reduces the chance of finding single-species-specific PCR primers for analysis. The combination of different cells types will contribute to a greater understanding of the PDAC-stroma cross-talk and advancing the field, ultimately, identifying new therapeutic targets. Our novel and well characterized 3D co-culture spheroid model of PDAC and stellate cells offers one piece to this complex puzzle. Future experiments utilizing the model will investigate the effects of the loss of epithelial characteristics in 3D co-culture, as well as the impact of different PDAC-PSC combinations on chemoresistance.

## Conclusion

We extensively characterized a scaffold-free, direct 3D co-culture spheroid model of human PDAC cells and PSCs. We also developed and validated a novel method for the investigation of cell type specific gene expression in direct cellular co-cultures of mixed species, without previous cell separation, termed virtual sorting. These models should greatly facilitate the examination of pancreatic tumour stroma cell crosstalk in 3D as well as the identification of novel PDAC vulnerabilities.

## Supplementary information

**Additional file 1: Figure S1.** Transmission electron microscopy of spheroid sections. Central sections giving an overview of representative HPAFII, Panc1 and hPSC monospheroids after 5 days of culture (a). The outer layer hPSCs from a day 3 PSC monospheroid have a flattened shape (arrow heads in b). A higher magnification Panc1/PSC heterospheroid from day 3 is shown with a vacuole (arrow) with inwards directed microvilli containing matrix (“M”) and collagen fibers (arrow heads, c). An HPAFII monospheroid from day 2 (d) showing cytoplasm with vacuoles (“V”) and glycocalyx-coated microvilli (arrow heads) as well as some matrix production (“M”).

**Additional file 2: Figure S2.** Expression analyses of Panc1/hPSC mono- and heterospheroids. mRNA expression of *CK19* (a*)*, *WT1* (b) and *CD10* (c) from spheroid cultures over a time period of 7 days, and normalized to the relative expression of the individual genes in Panc1 mono-spheroids/−cultures (MC) at day 3.

**Additional file 3: Figure S3.** Expression analyses of HPAFII/hPSC mono- and heterospheroids. Normalized mRNA expression of *CK19* (a) and *CD10* (b) from spheroid cultures over a time period of 7 days, and normalized to the relative expression of the individual genes in HPAFII mono-spheroids/−cultures (MC) at day 3.

**Additional file 4: Figure S4.** Immunohistochemical characterisation and expression analyses of HPAFII/hPSC mono- and heterospheroids. Immunohistochemical staining for VIM (a) and CD10 (b) of HPAFII and hPSC mono- and heterospheroids cultured for 5 days**.** mRNA expression of *ASMA*, *COL1A1*, *FN* and *TGFβ1* from spheroid cultures over a time period of 7 days are shown (c). All values have been normalized to the expression of the individual genes in HPAFII mono-spheroids/−cultures (MC) at day 3. A representative example of a CDH1 (E-cadherin; brown) protein staining from day 5 HPAFII and hPSC mono- and heterosphroids is shown together with a detailed quantification of CDH1-positive cells over a time period of 7 days (d). Black scale bars in a), b) and d) correspond to 100 μm.

**Additional file 5: Figure S5.** Relative growth curves for Panc1, HPAFII and hPSC mono- and heterospheroids. The maximal diameters were determined for Panc1 and hPSC mono- and heterospheroids (a) and HPAFII and hPSC mono- and heterospheroids (b). One representative of two experiments is depicted for each spheroid type and combination.

**Additional file 6: Figure S6.** Virtual sorting primers are species specific. Real time PCR products amplified with the indicated primers from human and mouse cell lines were analysed on a 2% agarose gel. The images show the original gels where also the loading slots and free primer are indicated, except for the gel in 6c, where the free primer has already run out of the gel. “h” indicates human and “m” mouse origin. Relevant sizes of a 100 bp molecular weight ladder run on each side of the samples are indicated by arrows. The calculated amplicon sizes are shown below each amplification. RPL13A/Rpl13a, housekeeping gene ribosomal protein 13a human/mouse.

**Additional file 7: Table S1.** Antibodies used for immunohistochemical analysis.

## Data Availability

All data generated or analysed during this study are included in this published article [and its supplementary information files].

## Abbreviations

3D: Three dimensional
ASMA: Alpha smooth muscle actin / ACTA2, actin alpha 2, smooth muscle
CC: Co-culture
CD10: Cluster of differentiation 10 / MME, membrane metalloendopeptidase
CDH1: Cadherin 1, E-cadherin
CK19: Cytokeratin 19
COL1A1: Collagen type I alpha 1 chain
ECM: Extracellular matrix
FN: Fibronectin
MC: Mono-culture
MKI67: Marker of proliferation
KPC: Kras^LSL-G12D/+^;Trp53^LSL-R172H/+^;Pdx-Cre
MCTS: Multicellular tumour spheroids
PBS: Phosphate buffered saline
PDAC: Pancreatic ductal adenocarcinoma
PSC: Pancreatic stellate cell
h: Human
m: Mouse
TEM: Transmission electron microscopy
TGFβ: Transforming growth factor-beta
VIM: Vimentin
WT1: WT (Wilms tumour) 1 transcription factor
