# Supplementary figures and images for: A novel pancreatic tumour and stellate cell 3D co-culture spheroid model
Source: BMC Cancer. 2020 May 27;20:475. doi: 10.1186/s12885-020-06867-5 (PMC7251727; doi:10.1186/s12885-020-06867-5)

## Slide 1
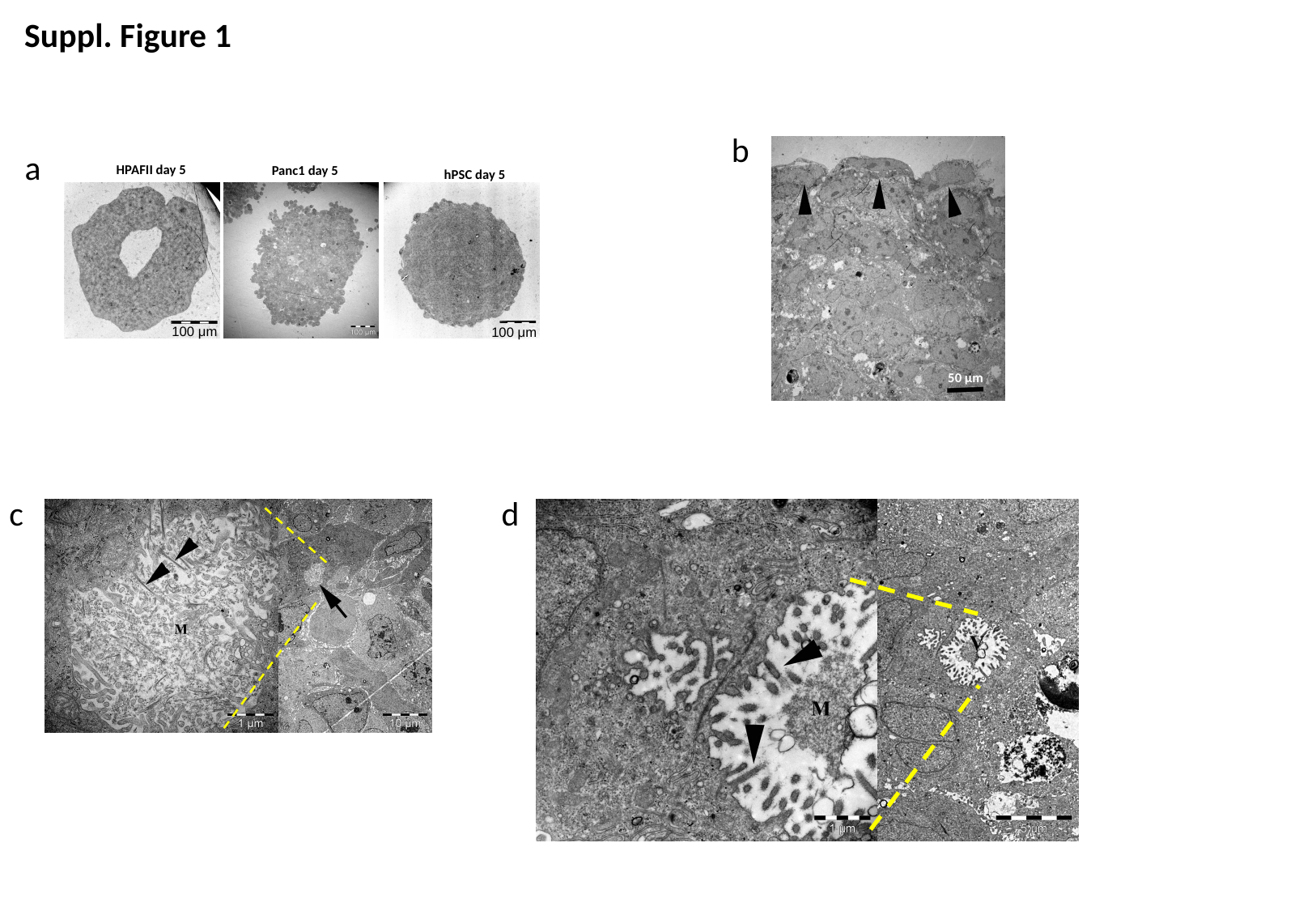

Suppl. Figure 1
b
50 µm
a
HPAFII day 5
Panc1 day 5
hPSC day 5
100 μm
100 μm
c
d

Supplement: Supplementary file 1 — Additional file 1: Figure S1. Transmission electron microscopy of spheroid sections. Central sections giving an overview of representative HPAFII, Panc1 and hPSC monospheroids after 5 days of culture (a). The outer layer hPSCs from a day 3 PSC monospheroid have a flattened shape (arrow heads in b). A higher magnification Panc1/PSC heterospheroid from day 3 is shown with a vacuole (arrow) with inwards directed microvilli containing matrix (“M”) and collagen fibers (arrow heads, c). An HPAFII monospheroid from day 2 (d) showing cytoplasm with vacuoles (“V”) and glycocalyx-coated microvilli (arrow heads) as well as some matrix production (“M”). [file 12885_2020_6867_MOESM1_ESM.pptx]

## Slide 1
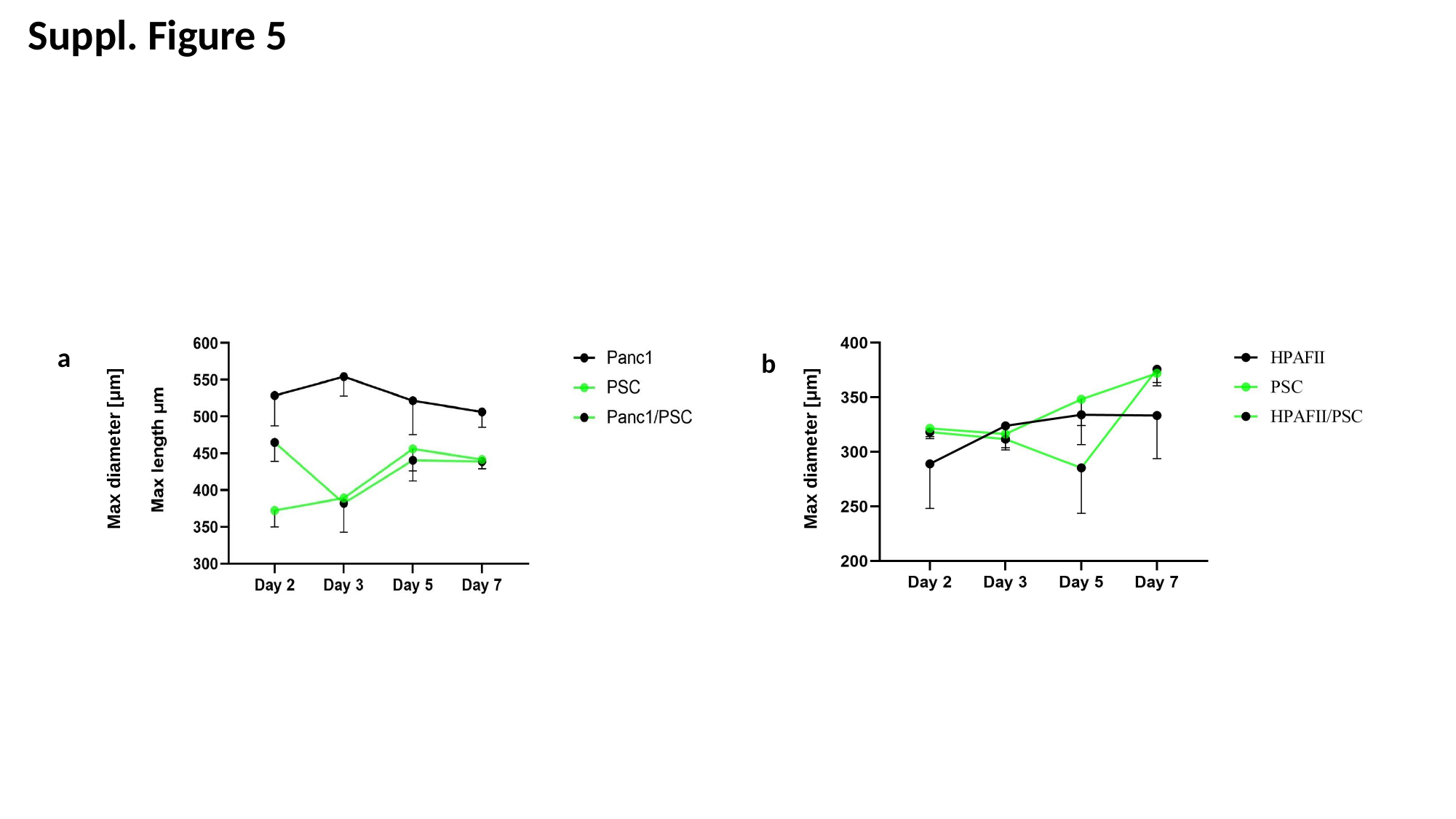

Suppl. Figure 5
Max diameter [μm]
Max diameter [μm]
a
b

Supplement: Supplementary file 5 — Additional file 5: Figure S5. Relative growth curves for Panc1, HPAFII and hPSC mono- and heterospheroids. The maximal diameters were determined for Panc1 and hPSC mono- and heterospheroids (a) and HPAFII and hPSC mono- and heterospheroids (b). One representative of two experiments is depicted for each spheroid type and combination. [file 12885_2020_6867_MOESM5_ESM.pptx]
