# Supplementary material for: A novel pancreatic tumour and stellate cell 3D co-culture spheroid model
Source: BMC Cancer. 2020 May 27;20:475. doi: 10.1186/s12885-020-06867-5 (PMC7251727; doi:10.1186/s12885-020-06867-5)
Supplement: Supplementary file 3 — Additional file 3: Figure S3. Expression analyses of HPAFII/hPSC mono- and heterospheroids. Normalized mRNA expression of CK19 (a) and CD10 (b) from spheroid cultures over a time period of 7 days, and normalized to the relative expression of the individual genes in HPAFII mono-spheroids/−cultures (MC) at day 3. [file 12885_2020_6867_MOESM3_ESM.pptx]

## Slide 1
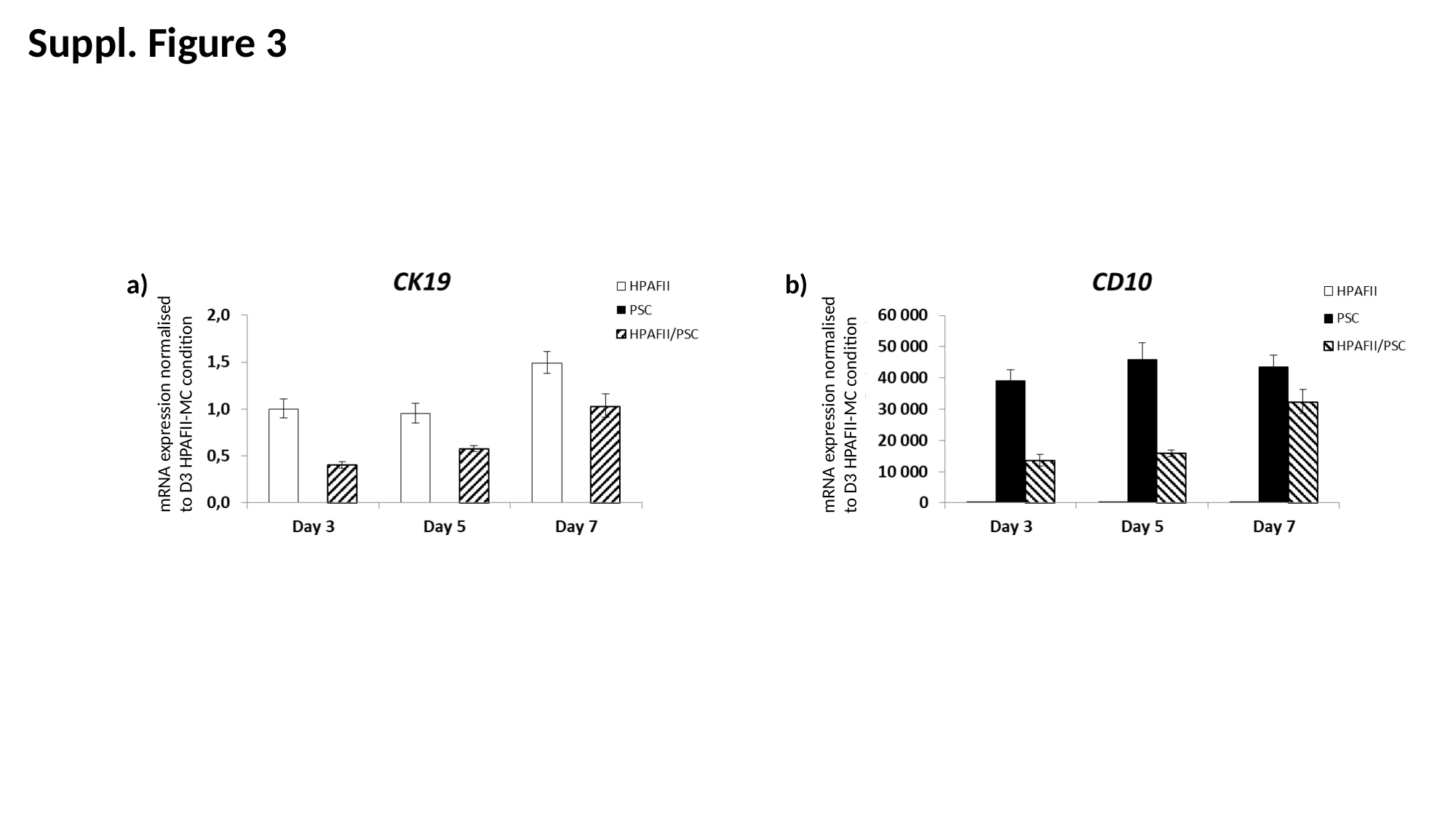

Suppl. Figure 3
mRNA expression normalised to D3 HPAFII-MC condition
a)
b)
mRNA expression normalised to D3 HPAFII-MC condition
