# Supplementary material for: A novel pancreatic tumour and stellate cell 3D co-culture spheroid model
Source: BMC Cancer. 2020 May 27;20:475. doi: 10.1186/s12885-020-06867-5 (PMC7251727; doi:10.1186/s12885-020-06867-5)
Supplement: Supplementary file 4 — Additional file 4: Figure S4. Immunohistochemical characterisation and expression analyses of HPAFII/hPSC mono- and heterospheroids. Immunohistochemical staining for VIM (a) and CD10 (b) of HPAFII and hPSC mono- and heterospheroids cultured for 5 days. mRNA expression of ASMA, COL1A1, FN and TGFβ1 from spheroid cultures over a time period of 7 days are shown (c). All values have been normalized to the expression of the individual genes in HPAFII mono-spheroids/−cultures (MC) at day 3. A representative example of a CDH1 (E-cadherin; brown) protein staining from day 5 HPAFII and hPSC mono- and heterosphroids is shown together with a detailed quantification of CDH1-positive cells over a time period of 7 days (d). Black scale bars in a), b) and d) correspond to 100 μm. [file 12885_2020_6867_MOESM4_ESM.pptx]

## Slide 1
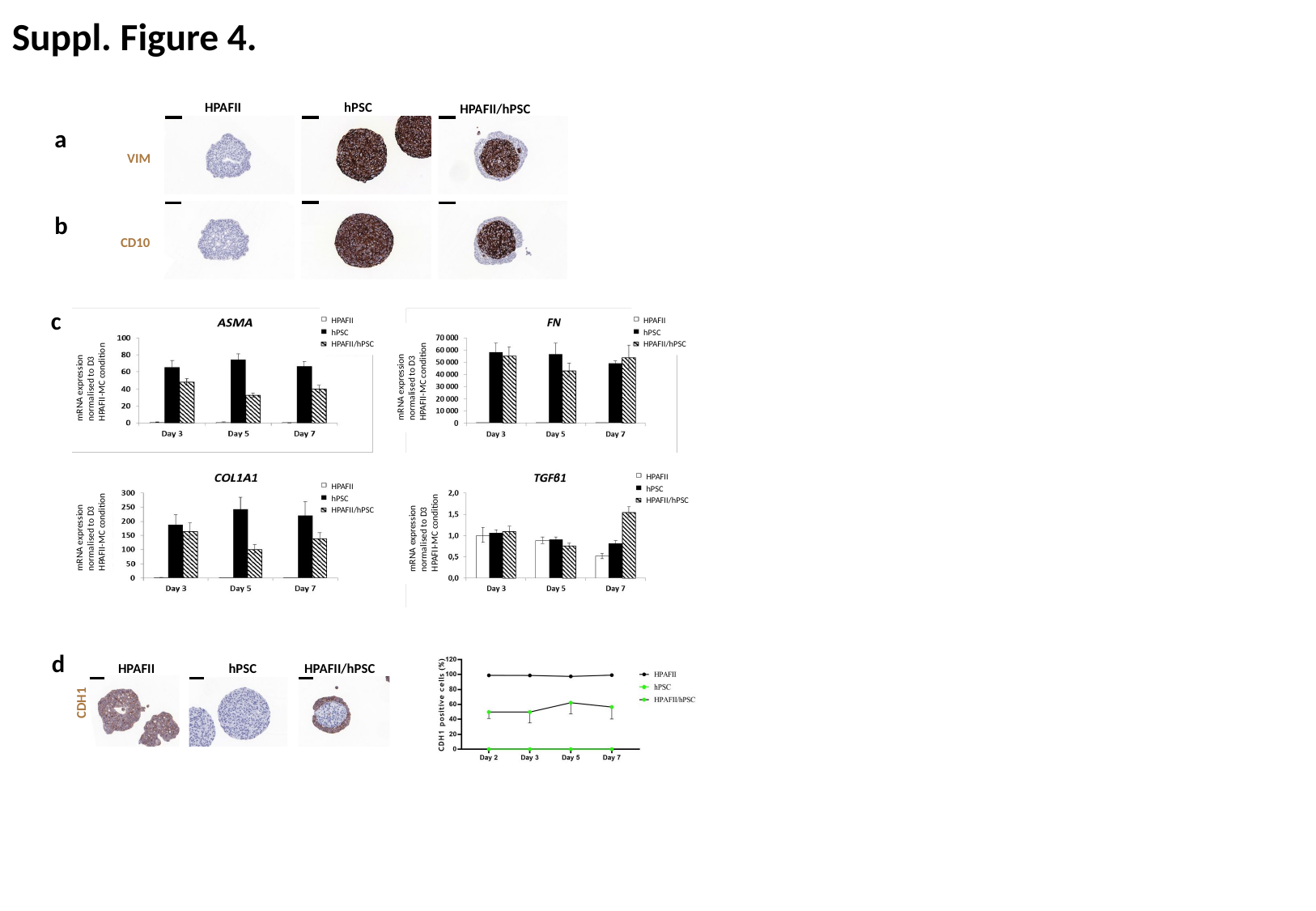

Suppl. Figure 4.
HPAFII
hPSC
HPAFII/hPSC
VIM
CD10
a
b
c
HPAFII
hPSC
HPAFII/hPSC
HPAFII
hPSC
HPAFII/hPSC
mRNA expression normalised to D3 HPAFII-MC condition
mRNA expression normalised to D3 HPAFII-MC condition
HPAFII
hPSC
HPAFII/hPSC
TGFβ
HPAFII
hPSC
HPAFII/hPSC
hPSC
mRNA expression normalised to D3
HPAFII-MC condition
mRNA expression normalised to D3 HPAFII-MC condition
d
HPAFII
hPSC
HPAFII/hPSC
CDH1
