# Supplementary material for: A novel pancreatic tumour and stellate cell 3D co-culture spheroid model
Source: BMC Cancer. 2020 May 27;20:475. doi: 10.1186/s12885-020-06867-5 (PMC7251727; doi:10.1186/s12885-020-06867-5)
Supplement: Supplementary file 6 — Additional file 6: Figure S6. Virtual sorting primers are species specific. Real time PCR products amplified with the indicated primers from human and mouse cell lines were analysed on a 2% agarose gel. The images show the original gels where also the loading slots and free primer are indicated, except for the gel in 6c, where the free primer has already run out of the gel. “h” indicates human and “m” mouse origin. Relevant sizes of a 100 bp molecular weight ladder run on each side of the samples are indicated by arrows. The calculated amplicon sizes are shown below each amplification. RPL13A/Rpl13a, housekeeping gene ribosomal protein 13a human/mouse. [file 12885_2020_6867_MOESM6_ESM.pptx]

## Slide 1
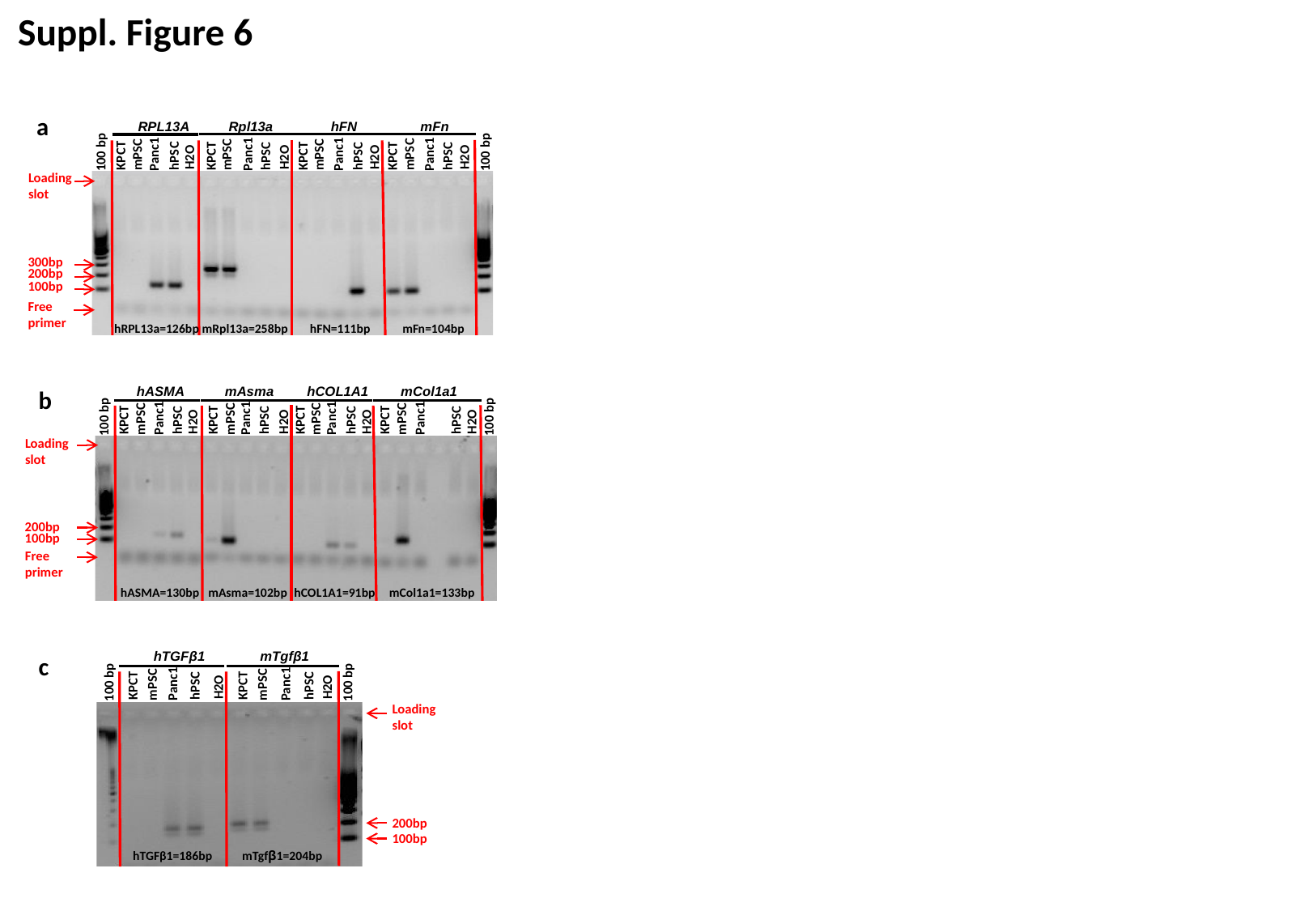

Suppl. Figure 6
a
hFN
Rpl13a
mFn
RPL13A
100 bp
100 bp
Panc1
Panc1
Panc1
Panc1
mPSC
mPSC
mPSC
mPSC
KPCT
KPCT
KPCT
KPCT
hPSC
hPSC
hPSC
hPSC
H2O
H2O
H2O
H2O
Loading
slot
300bp
200bp
100bp
Free
primer
hRPL13a=126bp
mRpl13a=258bp
hFN=111bp
mFn=104bp
hCOL1A1
mAsma
mCol1a1
hASMA
100 bp
100 bp
Panc1
Panc1
Panc1
Panc1
mPSC
mPSC
mPSC
mPSC
KPCT
KPCT
KPCT
KPCT
hPSC
hPSC
hPSC
hPSC
H2O
H2O
H2O
H2O
Loading
slot
200bp
100bp
Free
primer
hASMA=130bp
mAsma=102bp
hCOL1A1=91bp
mCol1a1=133bp
b
mTgfβ1
hTGFβ1
100 bp
100 bp
Panc1
Panc1
mPSC
mPSC
KPCT
KPCT
hPSC
hPSC
H2O
H2O
Loading
slot
200bp
100bp
mTgfβ1=204bp
hTGFβ1=186bp
c
