# Supplementary material for: A novel pancreatic tumour and stellate cell 3D co-culture spheroid model
Source: BMC Cancer. 2020 May 27;20:475. doi: 10.1186/s12885-020-06867-5 (PMC7251727; doi:10.1186/s12885-020-06867-5)
Supplement: Supplementary file 7 — Additional file 7: Table S1. Antibodies used for immunohistochemical analysis. [file 12885_2020_6867_MOESM7_ESM.pptx]

## Slide 1
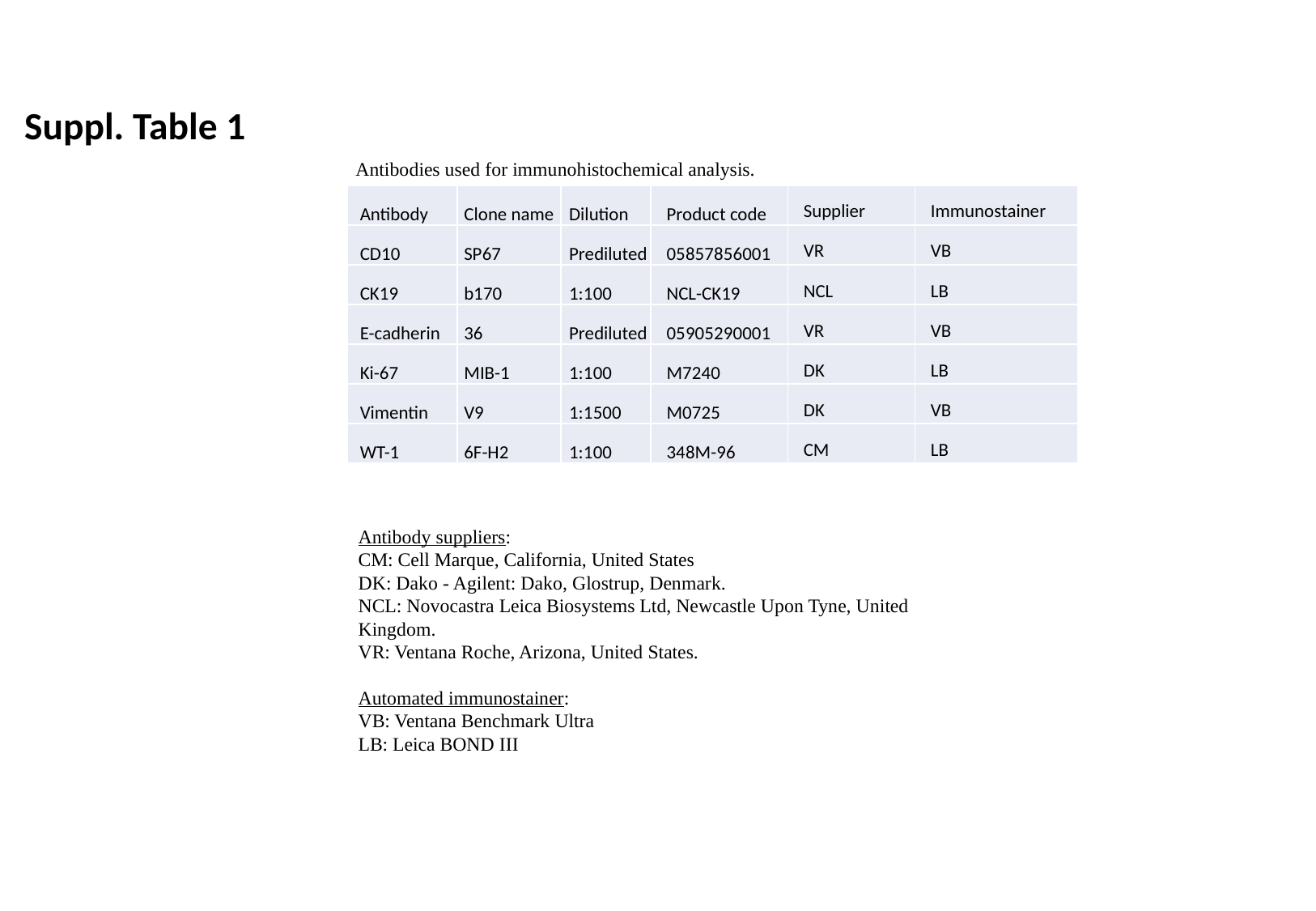

Suppl. Table 1
Antibodies used for immunohistochemical analysis.
| Antibody | Clone name | Dilution | Product code | Supplier | Immunostainer |
| --- | --- | --- | --- | --- | --- |
| CD10 | SP67 | Prediluted | 05857856001 | VR | VB |
| CK19 | b170 | 1:100 | NCL-CK19 | NCL | LB |
| E-cadherin | 36 | Prediluted | 05905290001 | VR | VB |
| Ki-67 | MIB-1 | 1:100 | M7240 | DK | LB |
| Vimentin | V9 | 1:1500 | M0725 | DK | VB |
| WT-1 | 6F-H2 | 1:100 | 348M-96 | CM | LB |
Antibody suppliers:
CM: Cell Marque, California, United States
DK: Dako - Agilent: Dako, Glostrup, Denmark.
NCL: Novocastra Leica Biosystems Ltd, Newcastle Upon Tyne, United Kingdom.
VR: Ventana Roche, Arizona, United States.
Automated immunostainer:
VB: Ventana Benchmark Ultra
LB: Leica BOND III
